# Dielectric Response of Quantum Critical Ferroelectric as a Function of Pressure

**DOI:** 10.1038/s41598-018-33320-2

**Published:** 2018-10-08

**Authors:** M. J. Coak, C. R. S. Haines, C. Liu, D. M. Jarvis, P. B. Littlewood, S. S. Saxena

**Affiliations:** 1 Cavendish Laboratory, Cambridge University, J. J. Thomson Ave, Cambridge, CB3 0HE UK; 2 Center for Correlated Electron Systems, Institute for Basic Science, Seoul, 08826 Republic of Korea; 3 Department of Physics and Astronomy, Seoul National University, Seoul, 08826 Republic of Korea; 4 Argonne National Laboratory, 9700 S. Cass Avenue, Lemont, Illinois 60439 United States; 5 James Franck Institute, University of Chicago, 929 E 57 St., Chicago, Illinois 60637 USA; 6 National University of Science and Technology “ MISiS”, Leninsky Prospekt 4, Moscow, 119049 Russia

## Abstract

In this work we report for the first time measurements of the dielectric loss of single-crystal SrTiO_3_ under the application of hydrostatic pressure up to 20 kbar and temperatures down to 200 mK which allow us to comment on the evolution of new fundamental material properties and their relationship with the recently discovered quantum critical phenomena in this material. The well known 18 K peak or shoulder was no longer observed after pressure was applied, even after subsequently removing it, suggesting it is associated with the twin walls formed at the 110 K cubic-tetragonal transition. The family of familiar peaks were all seen to increase in temperature linearly with pressure and the height of the 9.4 K peak was drastically suppressed by even the smallest pressures. This peak is discussed in the context of a postulated ferroelectric quantum critical point in SrTiO_3_ and the behaviour of its size linked to the position of this point on the recently established phase diagram.

## Introduction

Dielectric perovskite SrTiO_3_ has been long studied and discussed in great detail and, due to an anomalously high dielectric constant at low temperatures, finds use in many applications^1–7^. SrTiO_3_ has a cubic perovskite structure^8^ at room temperature, but an antiferrodistortive tilting transition of the oxygen octahedra occurs at 110 K^9^ to a tetragonal low-temperature phase. The dielectric constant, *ε*_*r*_, of SrTiO_3_ exhibits a classical Curie-Weiss temperature dependence at high temperatures^4^, but departs from the Curie-Weiss behaviour as the polarisation is modified by quantum fluctuations below 50 K and no ferroelectric ordering is observed. While long-range ferroelectricity does not form, a low-energy excitation of a soft transverse optical phonon mode is responsible for the very large polarisability and departures from classical predictions seen at low temperatures^5^. These phenomena have been linked to a postulated ferroelectric quantum critical point (QCP) at the equivalent of a small ‘negative pressure’ on SrTiO_3_’s pressure-temperature phase diagram^10,11^. Additionally, there is significant interest in the twin walls created at the cubic-tetragonal transition at 110 K as they are polar^12–14^ and ferroelastic^15^ and can be used to couple to other functional domain walls, in particular magnetic domain walls^16^ in heterostructures.

While the dielectric constant has been extensively measured, and several experiments using applied pressure to tune the phonon response of the system have been reported^3,11,17–19^, the out-of-phase component of the system’s polarisation, the dielectric loss, has only been reported in a handful of papers under ambient conditions and no reports could be found of studies of the loss under pressure. In particular Viana *et al*.^20^ contains the most comprehensive data of ambient-pressure dielectric loss in SrTiO_3_ using a similar capacitance measurement technique to that reported here and over a wide frequency regime including the 1 kHz used in this work. Ang *et al*.^21^ also report measurements of the loss and of the effect of bismuth doping upon it^22,23^, but many of their results were taken in the MHz frequency regime and may not be directly comparable. A strong loss peak at 10 K is reported by all authors, which Viana *et al*. observes to move downwards in temperature with decreasing frequency and also discusses in the context of a ferroelectric QCP. Also of interest is a peak in the lattice parameters of SrTiO_3_ at 10 K reported by Lytle^24^ from x-ray scattering. Additional features and peaks are reported by Viana *et al*. and Ang *et al*. at 16 K, 37 K and 65 K. The 37 K and 65 K peaks are explained by Scott^25^ to have energies assignable to identified optical phonon modes, the 37 K mode to an oxygen-only mode tied to oxygen deficiencies in the crystal and the 65 K to an out-of-phase oxygen octahedra rotation mode. The 16 K peak remains unexplained however and is in fact not observed in the work of Viana *et al*.

## Methods

We carried out high precision capacitance measurements on single crystal samples of SrTiO_3_ from Crystal GmbH with gold electrodes vacuum evaporated onto the surfaces in a parallel-plate capacitor geometry. Measurements under extremely hydrostatic pressure conditions were made possible by the development, in collaboration with CamCool Research Ltd, of a piston-cylinder clamp cell with miniature shielded coaxial cables running into the sample region and electrically isolated from the cell body. This eliminates stray capacitances from the wiring and allows picoFarad (pF) capacitance signals to be measured with stabilities of one part in a million. The shield conductors of the coaxial cables were joined together at the sample position and at the measurement instrument in the standard 2-point capacitance setup. An Andeen-Hagerling 2550A 1 kHz capacitance bridge was used, at a voltage of 0.1 V. Sample thickness, corresponding to capacitor plate separation, was 0.5 mm. Measurements were taken on a modified 1 K Dipper cryostat from ICE Oxford, allowing continuous stable temperature control down to 1.2 K and an adiabatic demagnetisation refrigerator developed in house capable of reaching 200 mK. Typical heating or cooling rates were held at 0.01 K per minute to allow the large thermal mass of the pressure cell to thermally equilibrate.

## Results

**Figure 1:**
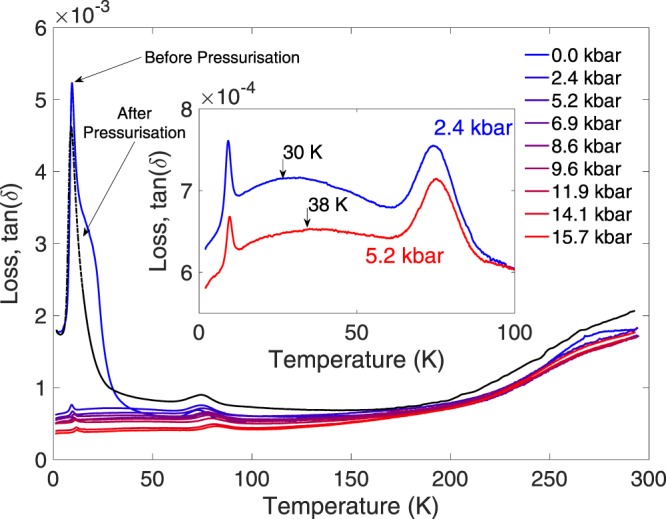
Dielectric loss of SrTiO_3_ plotted against temperature from zero applied pressure (topmost, blue) to 15.7 kbar (bottom, red) and with data taken at zero applied pressure after removing all the load in the cell shown in dashed black. The peak at 10 K has been drastically suppressed by even the lowest pressure applied and the act of pressurising the cell has permanently removed the feature at 16 K. Inset shows detail of the two pressure sweeps where a broad maximum around 30 K is most clearly seen.

Figure 1 shows the temperature dependence of the loss or dissipation factor $\tan (\delta )=\frac{{\rm{Resistive}}\,{\rm{Power}}\,{\rm{Loss}}}{{\rm{Capacitive}}\,{\rm{Power}}}$ for SrTiO_3_ at ambient pressure and at several applied pressure values up to 15.7 kbar. After these measurements were completed, all load was released gradually from the cell and it was measured again at zero applied pressure, but with the sample still inside the cell and pressure medium. The results from this measurement are displayed as a black dashed line in Fig. 1. As the measurements taken were by necessity AC (at 1 kHz), the dielectric loss represents the relative strength of both conduction processes and phase-shifted resonance effects. The dissipation factor comes from the imaginary or out of phase component of the dielectric constant *ε*_*r*_ + *iε*_*r*_′ and so the Kramers-Kronig relation would suggest that any features in the loss should be echoed by features in the capacitance at the same temperature and vice versa - but this only applies in the high-frequency regime. As shown in recent measurements^11^ no such changes in *ε*_*r*_ could be resolved at the positions of loss peaks despite the high levels of precision used.

At ambient pressure a strong peak is seen at 9.4 K with a shoulder around 18 K, as well as a smaller peak at 73 K. A broad peak can also be resolved in some data sets centred on 30 K at low pressures and increasing as pressure is raised. Previous work by Viana *et al*.^20^ and Ang *et al*.^21^ has reported a peak at 10 K as well as anomalies seen in both the dielectric and ultrasonic responses at 16, 37 and 65 K. These anomalies were reported in both single crystal and ceramic samples and have been linked to oxygen vacancy defects^25^. It should be noted that all of these peaks are frequency-, as well as temperature-dependent, as described by Viana *et al*., so the absolute temperature positions and heights of each peak are dependent on the frequency used. Either temperature or frequency can be viewed as an experimental parameter in investigating the dielectric loss; the change of the temperature-dependence of the loss with pressure at a fixed frequency described here remains both valid and significant. The mismatch seen between the data in Fig. 1, which have been verified across several samples and the results in Ang *et al*. could be explained by the different frequencies used (1 kHz in this work and frequencies in the MHz range for the previous data), or potentially by sample preparation differences. The work of Venturini *et al*.^26^ also reports an equivalent 10 K peak in SrTi^18^O_3_ and notes a strong suppression of it at the ferroelectric QCP. This work additionally compares measurements of a potentially multidomain sample within a pressure cell environment with those on a carefully prepared monodomain crystal and find no significant differences - allowing us to feel confident in disregarding such complications.

The broad shoulder in the low temperature peak at around 18 K can be identified as the 16 K anomaly in the literature and the peak at 73 K as the 65 K anomaly. The 37 K feature, reported to correspond to a freezing of domain walls, can be attributed to the broad maximum shown in the inset to Fig. 1. It should also be noted that anomalies in the elastic response of SrTiO_3_ were reported around 80 K and 40 K by Nes *et al*.^27^.

The effect of applied pressure on the dielectric loss is shown in Fig. 1. Even the smallest pressure applied drastically suppresses the peak at 10 K and and the feature at 18 K is no longer seen in any high pressure data. Additionally, upon removing the pressure and measuring again (black dashed line in Fig. 1), the 18 K shoulder is no longer seen. Although the pressure inside such a piston cylinder cell is close to ideally hydrostatic, strains and preferred directions may be induced inside the sample, as well as the voltage applied during repeated cooling and warming cycles. The 18 K shoulder can therefore be most reasonably attributed to a twinning or domain effect within the crystal, which is then absent after the sample has been de-twinned by the application of pressure.

Viana *et al*.^20^ explain the observed 10 K peak in terms of a quantum phase transition and possible unconventional quantum ferroelectric states and observe that increasing frequency raises the peak height and shifts it downwards in temperature as well as a much larger peak at 70 K than described here. The enhancement of the 10 K peak as the QCP is approached with decreasing pressure suggests it is indeed linked to the quantum critical nature of the system. The overall trend across the whole temperature range is a reduction of the loss with pressure, as previously reported at room temperature by Samara and Giardini^28^, although the low values below 10^−3^ in this work suggest a better quality sample and measurement conditions than in the reference. The overall magnitude of the loss and its temperature variation are very sensitive to stray impedances and noise in the experimental setup and wiring due to the very small signal from the highly insulating sample - the reproducibility and clear trend in the pressure data is evidence to the stability of conditions within the pressure cell. The ambient pressure data not matching the trend of the data at high pressure is likely due to some such artefact.

**Figure 2:**
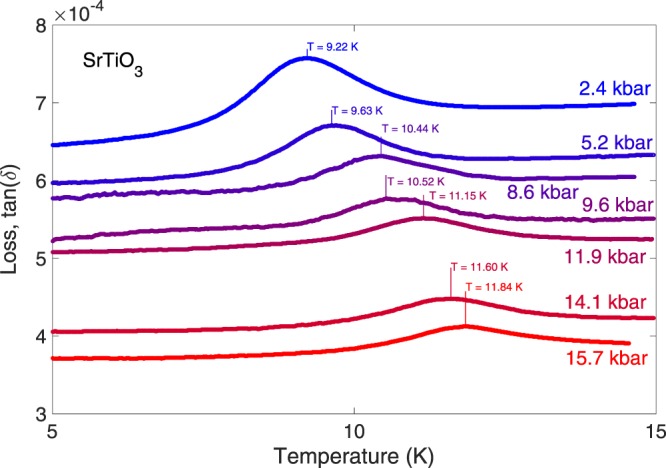
Detail of the 10 K peak in the loss of SrTiO_3_ for pressures from 2.4 (topmost, blue) to 15.7 kbar (bottom, red) - the ambient pressure peak is too large for ease of comparison.

**Figure 3:**
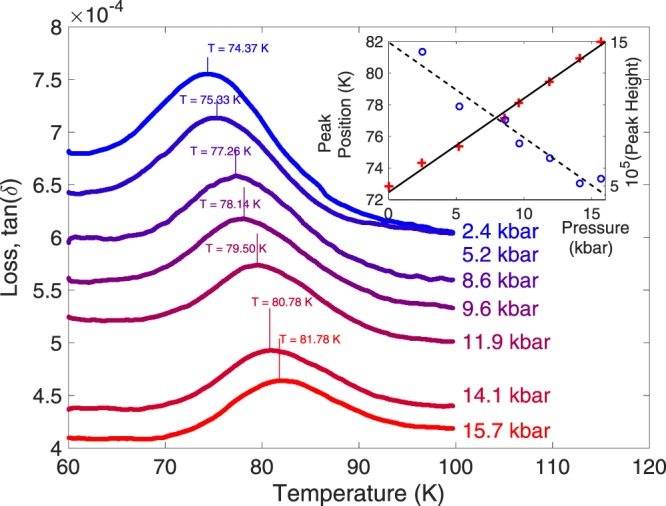
Detail of the 70 K peak in the loss of SrTiO_3_ for pressures from 2.4 kbar (topmost, blue) to 15.7 kbar (bottom, red), normalised over the range shown for ease of comparison. Inset shows the peak temperature position (red crosses) shifting linearly up with pressure with slope 0.60 Kkbar^−1^ and the peak height (blue circles) decreasing linearly with pressure.

**Figure 4:**
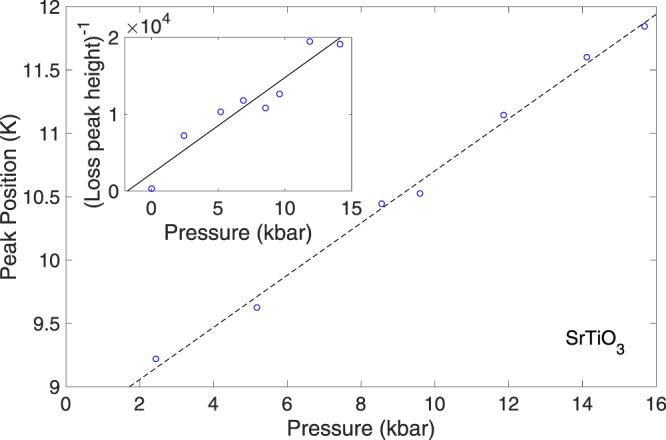
Temperature and inverse peak height (inset) of the 10 K peak in the dielectric loss of SrTiO_3_ plotted against pressure.

Figures 2 and 3 show the pressure evolution of the temperatures of the 10 K and 70 K peaks in the dielectric loss of SrTiO_3_. Both peaks move upwards in temperature as pressure is increased, suggesting an increase in the energy scale of the effects responsible, but this energy scale does not collapse to zero at the inferred QCP critical pressure. As with many effects presented here in SrTiO_3_, both shifts are linear in pressure, suggesting that the pressure ranges used act only as a small perturbation to the overall system - exactly the conditions desired to study the tuning of quantum critical properties. The 10 K peak is shifted by 0.21 Kkbar^−1^ (Fig. 4), the 70 K by 0.60 Kkbar^−1^ (Fig. 3 inset, red crosses). As the 70 K and 30 K peaks can be attributed to certain phonon modes as discussed in the introduction^25^ and the phonon frequencies are known to increase linearly with applied pressure^29^, the temperatures of these two peaks would be expected to increase linearly with pressure as observed. The magnitude of the 70 K peak decreases linearly with pressure (Fig. 3 inset, blue circles), consistent with the overall reduction in the magnitude of the signal. The striking result of the large 10 K peak height in only the ambient pressure measurement can be explained by plotting the reciprocal of the peak height against the applied pressure, shown in the inset of Fig. 4. Given the uncertainties arising from the noisy and complex nature of the loss as discussed, the fit to a linear pressure dependence is sufficient to suggest a Δ(tan(*δ*)) ∝ (*p* − *p*_*c*_)^−1^ relationship for the peak height. This analysis leads to a critical pressure *p*_*c*_ of −2.0 ± 2.0 kbar. This value of *p*_*c*_ is consistent with the critical pressure of a projected QCP reported in our recent work^11^, found from the dielectric constant measurements. The error in the peak height, including the broadening expected, based on Viana’s results, from its temperature shift (estimated 10%) and the number of data points determine the uncertainty in *p*_*c*_. Our measurements are therefore consistent with the 10 K peak in the loss being an effect resulting from the quantum critical state of SrTiO_3_. The increase in peak height in proximity to the quantum critical point and ferroelectricity is then explained by the divergence of the correlation length of ferroelectric fluctuations, leading to an increasing phase-shifted excitation peak in the loss.

**Figure 5:**
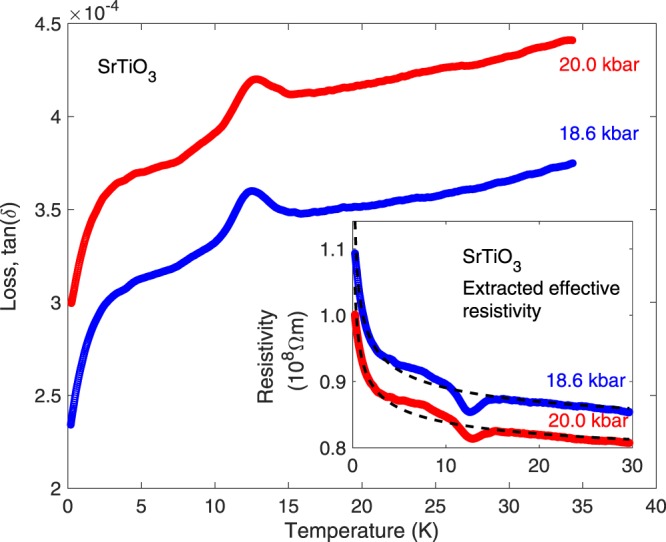
Loss of SrTiO_3_ down to 200 mK at 18.6 kbar (bottom, blue) and 20.0 kbar (upper, red). Inset shows an effective resistivity extracted from these data as described in the text, with dotted lines showing a fit to a 3D variable-range-hopping expression. Besides the prominent features at 10 K, the background loss follows this trend closely over more than two decades of temperature.

Measurements down to 200 mK at the two highest pressures revealed a downturn in the loss at the lowest temperatures (Fig. 5). When plotted on a logarithmic temperature axis the data become straight lines, aside from the 10 K excitation feature, furthermore, multiplying the loss data with their corresponding capacitance data sets allows an effective AC conductivity and resistivity to be calculated:

1 $$\tan (\delta )=\frac{\sigma }{{\varepsilon }_{r}{\varepsilon }_{0}\omega }$$

2 $$\rho =\frac{1}{\sigma }=\frac{1}{{\varepsilon }_{r}{\varepsilon }_{0}\omega \,\tan (\delta )}$$

Resistivity *ρ* extracted from the data in this manner, plotted in the inset of Fig. 5, was found to fit best to a three-dimensional variable-range-hopping^30^ expression $\rho \propto \exp ({(\frac{{T}_{0}}{T})}^{\mathrm{1/4}})$, outside the regime around the 10 K peak where conductivity is not dominating the loss. As Viana *et al*. noted^20^, the temperature dependence of the loss is of an activated exponential form, but the simple Arrhenius form does not adequately describe the data. Though we hesitate to interpret effective resistances of this immense magnitude as true electrical conduction, we nonetheless observe this clear temperature dependence of the loss over more than two decades of temperature. A puzzle is then the tiny extracted band gaps of 9.6 and 7 *μ*eV and that the variable-range-hopping model is formulated in terms of a highly-disorded local environment for the carriers, while all other data suggest a very high purity crystal. Interestingly however, measurements of the resistivity of SrTiO_3_ thin films by Li *et al*.^31^ with carriers doped into the sample via electrostatic gating exhibit a (two-dimensional due to the thin film sample) variable-range-hopping temperature dependence, in striking agreement with that observed here in this indirect method.

## Discussion

The dielectric loss of single-crystal SrTiO_3_ was measured via a capacitance method at 1 kHz and 0.1 V as a function of temperature and hydrostatic pressure. Peaks were seen in the ambient-pressure data at 9.4 K (with a shoulder around 18 K), 30 K and 73 K. These were attributed to the peaks previously reported by Ang *et al*., Venturini *et al*. and Viana *et al*.^20,21,26^ at 10, 16, 37 and 65 K. The 18 K peak or shoulder was not present in any measurements taken under applied pressure, and upon removing the cell’s load and measuring again it is also no longer seen. The 18 K shoulder was therefore attributed to a twinning or domain effect within the crystal, which is removed by the application of pressure. Previous literature has provided strong evidence for a ferroelectric quantum critical point (QCP) in SrTiO_3_ at the tuning equivalent of an effective small negative pressure and our recent work has shown multiple energy scales of the system collapse to zero at a projected critical pressure *p*_*c*_ of −0.7 ± 0.1 kbar^11^. The 9.4 K and 73 K peaks move upwards in temperature linearly as a function of applied pressure is increased (the 30 K peak also appears to increase linearly in temperature but was not clearly resolved in enough data to be conclusive). The temperature scale of the 9.4 K peak, a peak attributed by Viana *et al*. to quantum critical effects does not collapse to zero at the inferred QCP critical pressure; however the magnitude of the peak appears to be linked to the QCP, in agreement with Venturini’s results. The peak dramatically decreases in height as the system is tuned away from the critical point and plotting the reciprocal of the peak height against pressure yields a straight-line relation and a *p*_*c*_ of −2.0 ± 2.0 kbar. This value agrees, within error, with the *p*_*c*_ of −0.7 ± 0.1 kbar found from the real component of the dielectric constant in our recent report^11^. We suggest this peak to reflect a localized excitation formed of quantum fluctuations of domain walls - the temperature or frequency of the excitation is then related to the barrier between the ‘up’ and ‘down’ polarisation states and hence does not collapse to zero at the QCP. The amplitude of the peak, however, is then given by the number of such fluctuations and hence grows as the QCP is approached.

The temperature of the 73 K peak increased linearly with pressure, consistent with the explanation of Scott^25^ that this peak is linked to an optical phonon mode, shown^29^ to increase in energy linearly as a function of pressure. The height of the peak was decreased linearly with pressure, in line with the overall reduction of the magnitude of the dielectric loss. In agreement with the data of Viana *et al*., the background loss value, excluding peaks, measured down to 200 mK showed an exponential activation-type temperature dependence, but was not well described by the simple Arrhenius form. A variable-range hopping expression, typically used to describe highly-disordered systems but also observed in gated SrTiO_3_ thin films, was found to fit the data the best, with very small *μ*eV effective gaps, potentially caused by impurity or oxygen deficiency bands, but may not reflect a true electrical resistance but another effect such as domain wall motion governed by some kind of activated response.

## Data Availability

The datasets generated during and/or analysed during the current study are available from the corresponding authors on reasonable request.
